# Fabrication of a Novel Antifouling Polysulfone Membrane with in Situ Embedment of Mxene Nanosheets

**DOI:** 10.3390/ijerph16234659

**Published:** 2019-11-22

**Authors:** Zhen Shen, Wei Chen, Hang Xu, Wen Yang, Qing Kong, Ao Wang, Mingmei Ding, Juan Shang

**Affiliations:** 1 Key Laboratory of Integrated Regulation and Resource Development on Shallow Lakes, Ministry of Education, College of Environment, Hohai University, No.1 Xikang Road, Nanjing 210098, China; 160205010001@hhu.edu.cn (Z.S.); cw5826@hhu.edu.cn (W.C.); 181305010022@hhu.edu.cn (W.Y.); 181305010017@hhu.edu.cn (Q.K.); 1714040327@hhu.edu.cn (A.W.); 170405010001@hhu.edu.cn (M.D.); 2 Wanjiang University of Technology, Maanshan 243031, China; sz_jane_207@126.com

**Keywords:** MXene (Ti_3_C_2_T_x_), polysulfone, hydrophilicity, ultrafiltration membrane, antifouling

## Abstract

Membrane fouling is still a critical issue for the application of ultrafiltration, which has been widely used in water treatment due to its efficiency and simplicity. In order to improve the antifouling property, a new 2D material MXene was used to fabricate composite ultrafiltration membrane with the approach of in situ embedment during the phase inversion process in this study. Scanning electron microscopy (SEM), atomic force microscopy (AFM), thermogravimetric analysis (TGA), energy dispersive spectroscopy (EDS), Fourier transform infrared spectroscopy (FTIR), water contact angle, bovine serum albumin rejection and porosity measurements were utilized to characterize the prepared membranes. Due to the hydrophilicity of the MXene, the composite membranes obtained higher hydrophilicity, confirmed by the decreased water contact angle. All the modified membranes had a high bovine serum albumin rejection above 90% while that of the pristine polysulfone membrane was 77.48%. The flux recovery ratio and the reversible fouling ratio of the membranes were also improved along with the increasing content of the MXene. Furthermore, the highest flux recovery ratio could also reach 76.1%. These indicated the good antifouling properties of MXene composite membranes. The enhanced water permeability and protein rejection and excellent antifouling properties make MXene a promising material for antifouling membrane modification.

## 1. Introduction

Ultrafiltration (UF) technology is effective for turbidity and pathogen removal at low pressures, so it has been widely used in the process of drinking water treatment. The advantages of UF include high water production, compact modular design, and good adaptation to changes in water quality [1,2]. Owing to these advantages, UF membrane systems are now widely applied in water plants in order to simplify the conventional water treatment process. Polysulfone (PSF), as a resin material, is now widely used as the raw material for ultrafiltration membrane fabrication due to its outstanding mechanical properties, great chemical resistance, good thermal stability and wide pH operation range. However, the hydrophobic nature of polysulfone could result in membrane fouling which may reduce membrane service life and thus has become a major challenge for its wide applications.

Membrane modification such as chemical grafting, surface modification or physical doping can overcome the weaknesses caused by the hydrophobic nature of PSF and this has attracted much attention [3]. Among various modification methods, physical blending of PSF with nanomaterials has gained great popularity for its ease, cost-effectiveness and ability to retain the primary morphology of the membrane [4,5]. Ding et al. [6] fabricated the ultrafiltration membrane using N-doped graphene oxide (NRG)/TiO_2_ as a filler, showing good improvement of hydrophilicity, fouling resistance and permeability for the PSF membrane. Khan et al. [7] used modified nanocarbon black as nanofillers to enhance the antifouling properties of the ultrafiltration membrane, and the results showed that the highest flux recovery reached up to 89.4% after three cycle tests. Therefore, finding a suitable inorganic material has become a hot spot in current research.

Recently, a new family of 2D materials named “MXene” has received great attention in the field of membrane separation [8,9,10]. MXenes are a type of layered 2D materials prepared by removing the A layer of their precursor MAX phases, where M represents the early transition metal, A corresponds to the group element (mainly IIIA or IVA group), and X stands for C and/or N. The MXene defined as M_n+1_X_n_T_x_, where T refers to the function groups (e.g., O, F or OH). It has hydrophilic surfaces, good structure, great chemical stability as well as excellent electrical conductivity [11,12,13]. Owing to these advantages, MXene has been widely utilized in the field of separation membranes for water desalination, gas separation, ion sieving, and wastewater treatment [14,15,16,17,18]. Meanwhile, considerable attention has been paid to adding carbon-based nanomaterials into polymer to fabricate ultrafiltration membranes. Compared to normal polymer membranes, these modified membranes have obvious improvements in hydrophilicity, mechanical strength, as well as antifouling ability [19,20,21]. Han et al. fabricated a new 2D MXene/PES composite membrane, showing excellent hydrophilicity and enhanced water flux and high rejections to Congo red dye [11]. However, there are few studies on the antifouling ability of MXene membranes.

In this work, MXene (Ti_3_C_2_T_x_) were incorporated into PSF membrane to fabricate a new kind of nanocomposite membrane, which has an improved hydrophilicity and antifouling performance. Selective etching followed by delaminating of Ti_3_C_2_T_x_ powders was utilized to synthesize MXene nanosheets. The resulting MXene nanosheets were dispersed in water and were incorporated into PSF membrane by the in situ embedment method. The effect of different MXene concentrations on hydrophilicity, surface charge, porosity and pore structure were systematically evaluated. The morphology, water permeation, separation performance and antifouling properties of the composite membranes were studied following the standard experimental protocol.

## 2. Materials and Methods

### 2.1. Materials

HCl (36–38%, AR) was bought from Lingfeng Chemical Reagent, China. LiF (99.9%) was purchased from Aladdin Industrial Corporation, Shanghai China. MAX phase (Ti_3_AlC_2_) which was utilized for chemical etching to obtain MXene nanosheets was purchased from Ming Shan new materials corporation, Nanjing China. N-methylpyrrolidone (NMP, AR), polyvinylpyrrolidone (PVP, GR), polysulfone (PSF, AR) and bovine serum albumin (BSA, MW = 67,000 g/mol) were purchased from China Sinopharm International (Shanghai, China) Corporation.

### 2.2. Synthesis of MXene Nanosheets

**Figure 1:**
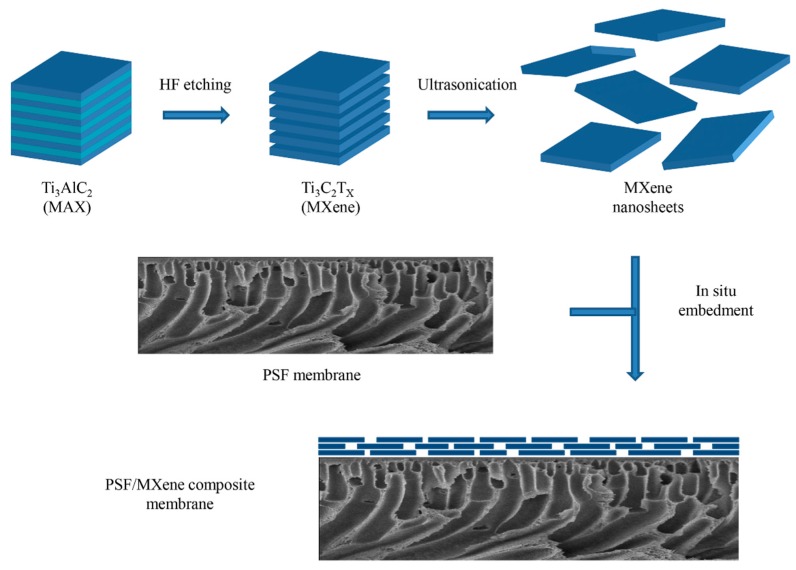
Schematic illustrations of the preparation and embedment of the MXene nanosheets.

The schematic of synthesis and embedment of MXene nanosheets is shown in Figure 1. In this work, the precursor used to synthesis MXene nanosheets was Ti_3_AlC_2_. It has a brick-like structure with firmly stacked layers which is hard to delaminate by sonication because of the Ti-Al metallic bond [22]. Therefore, in situ HF formation was utilized to exfoliate Ti_3_AlC_2_ powders to obtain the Ti_3_C_2_T_x_ MXene powders. Then, the MXene nanosheets were produced by sonication.

In brief, 1.0g LiF was added into a plastic beaker with 20mL of 9M HCl with stirring at room temperature for 5 min so that the etchant could be prepared. Then 1.0g Ti_3_AlC_2_ powders were gradually added to the etchant in 10 min with magnetically stirring and 24h was needed to complete the reaction. The resulting suspension was washed with deionized (DI) water via centrifugation for several cycles (5 min per cycle at 3500 rpm) until pH 5 was achieved. Afterwards, bottom sediment was diluted with 100mL DI water for ultrasonication delamination for 1h. Then Ti_3_C_2_T_x_ nanosheets would be finally obtained after centrifugation for 1h [16,23,24].

### 2.3. Membrane Fabrication and in Situ Nanosheet Embedment

Amounts of 17 wt % PSF and 3 wt % PVP additive were dissolved in NMP (80 wt %) with continuous stirring at 150 rpm of 50 °C for 8 h. Then, the beaker with casting solution was placed in water bath cauldron for 24 h in order to remove air bubbles. Afterwards, the solution was cast onto a clean and flat glass plate to form a film with a self-made casting knife. The film on the glass plate was then immersed into a water bath at room temperature.

According to the literature [25], the water bath used for membrane formation was dispersed with the MXene nanosheets at a certain concentration (0mg/L, 100mg/L, 300mg/L, 500mg/L, respectively) to embed MXene nanosheets. Correspondingly, the fabricated membranes are represented by M0, M1, M2 and M3, respectively.

### 2.4. Characterization of Membranes

The morphologies of the composite membranes were observed by using scanning electron microscopy. Furthermore, the surface roughness of the membrane was investigated by atomic force microscope. Energy dispersive spectroscopy (EDS; S-4200N, Hitachi, Tokyo, Janpan) was utilized to determine the quality of the dispersion and the existence of MXene nanosheets on the membrane surface. Fourier transform infrared spectroscopy (FTIR; Nicolet 6700, Thermo Electron Corp., Madison, WI, USA) was also applied to characterize the membranes.

To measure the thermal stability of the membranes, thermogravimetric analysis (TGA; 409pc, Netzsch, Selb, Germany) was applied with a heating rate of 10 °C/min from 20 °C to 800 °C under inert atmosphere.

The hydrophilicity of the membranes was evaluated from the water contact angle. Further, the water contact angle of membranes was measured with a contact angle goniometer at room temperature (G10, Kruss, Hamburg, Germany). Besides, the membrane surface charge was also tested by streaming potential measurement using a SurPASS analyzer (Anton Paar Gmbh, Austria).

The membrane porosity (ε) was calculated according to Equation (1) [26]

(1) $$\varepsilon \left(\%\right)=\frac{\frac{{(W}_{W}{-\;W}_{D})}{\rho_{W}}}{\frac{{(W}_{W}{-\;W}_{D})}{\rho_{W}}\;+\;\frac{W_{D}}{\rho_{P}}}\times 100$$

where W_W_ is the weight of wet membranes (g), W_D_ is the weight of dry membranes (g), ρ_w_ is the density of pure water at operating conditions (g·cm^−3^), and ρ_p_ is the density of the polymer (g·cm^−3^).

Mean pore size (r_m_) was calculated by the Guerout–Elford–Ferry method in Equation (2):

(2) $$r_{m}=\sqrt{\frac{\left(2.9-1.75\cdot \varepsilon \right)\cdot (8\cdot \eta \cdot l\cdot j)}{\varepsilon \cdot A_{m}\cdot \Delta P}}$$

where η stands for the water viscosity (8.9·10-4 Pa·s); ∆P represents the operation pressure (0.1MPa); l is the thickness of membrane (m); ε is the membrane porosity, A_m_ is the effective membrane area (m^2^) and j is the volume of permeate water per unit time (L/s) [26,27].

### 2.5. Water Permeability and Separation Performance Tests

Water permeability and separation performance of the prepared membranes were evaluated through laboratory scale self-made dead-end filtration equipment with a valid membrane area of 31.16 cm^2^. First, each membrane was compacted at 0.2 MPa with pure water for 30 min to obtain a steady flux. Following this, the pressure was reduced to the operation pressure of 0.1 MPa to test the water flux. The water flux was calculated with the following equation:

(3) $$J_{w,1}=\frac{M}{A\cdot \Delta t}$$

where M is the weight of the permeated pure water (kg), A is the membrane effective area (m^2^) and ∆t is the permeation time (h).

After that, an aqueous feed BSA solution was prepared with the concentration of 500mg/L to evaluate the performance of ultrafiltration. Rejection (R) at any point in the filtration process was defined as follows:

(4) $$R\left(\%\right)=\left(\frac{C_{F}{-C}_{P}}{C_{F}}\right)\;\times \;100\%$$

where C_P_ is the BSA concentration in the permeation and C_F_ is that in the feed solution.

### 2.6. Antifouling Performance Evaluation

The static protein adsorption on membrane was evaluated by solution depletion method. Bovine serum albumin (BSA) was used as model foulants. The prepared membranes with a diameter of 2 cm were immersed in 1g/L BSA solution prepared in phosphate buffer saline (PBS, pH 7.4) at 25 °C for 24 h. The concentration difference before and after adsorption was measured by an ultraviolet spectrophotometer at 280 nm. The result could represent the adsorption capacity of each membrane.

After water permeability and separation performance tests, the fouled membranes were washed with pure water for 15 min to clean the membranes contaminated by protein. Afterwards, the water flux was measured again as J_w, 2_(L·m^−2^·h^−1^). The flux recovery ratio (FRR) was defined as follows [28]:

(5) $$\mathrm{FRR}=\left(\frac{J_{w,2}}{J_{w,1}}\right)\times 100\%$$

In order to analyze the fouling process more accurately, the total fouling ration (R_t_) with reversible fouling ratio (R_r_) and irreversible fouling ratio (R_ir_) was used and calculated by the following equations [28,29]:

(6) $$R_{t}\left(\%\right)=\left(1-\frac{J_{p}}{J_{w,1}}\right)\times 100\%$$

(7) $$R_{r}\left(\%\right)=\left(\frac{J_{w,2}{-J}_{p}}{J_{w,1}}\right)\times 100\%$$

(8) $$R_{\mathrm{ir}}\left(\%\right)=\left(\frac{J_{w,1}{-J}_{w,2}}{J_{w,1}}\right)\times 100\%$$

## 3. Results and Discussions

### 3.1. Morphology and Structure of the Membranes

**Figure 2:**
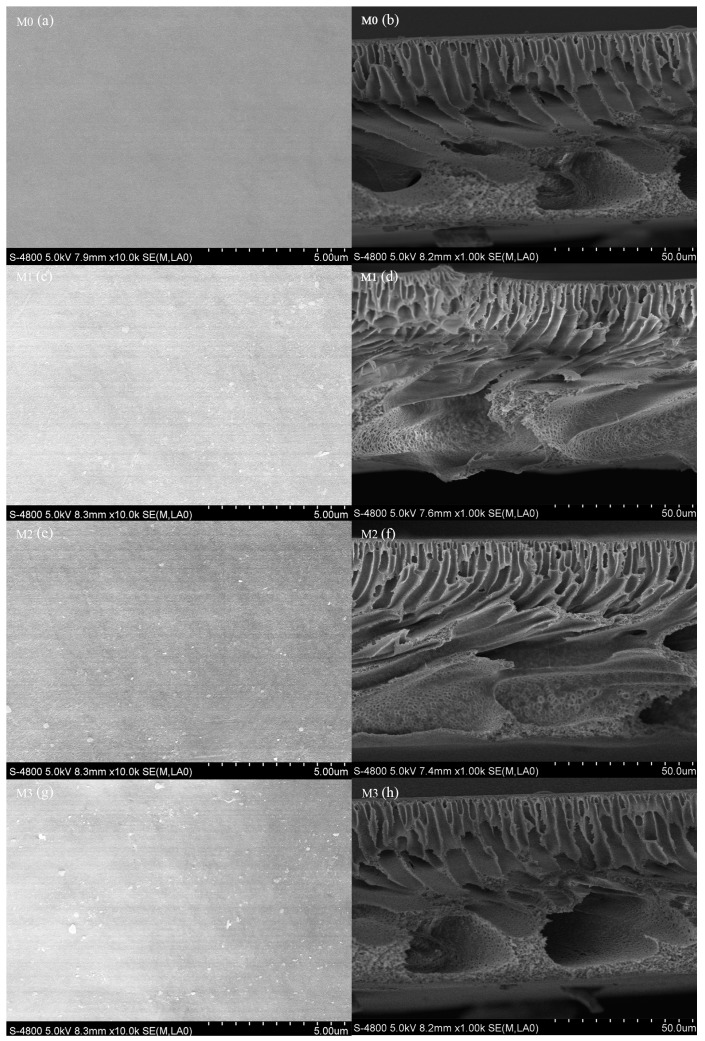
SEM images of the prepared membranes: M0 (**a**,**b**); M1 (**c**,**d**); M2 (**e**,**f**); M3 (**g**,**h**).

SEM was utilized to characterize the surface and the cross-section of the composite membrane. Figure 2 shows the top surface of all the membranes and MXene nanosheets as can be seen on membranes M1, M2, and M3. The MXene nanosheets were well dispersed in the polymer matrix due to their carbon based structure [11,12,30]. In addition, there were no cracks on the surface, contributing to the good stability of the membrane.

Figure 2 also exhibits the cross-sectional SEM images of the prepared membranes with different MXene contents. All of the membranes had a typical asymmetric structure which consisted of a finger-like porous sublayer and a dense top layer [30]. During the phase inversion process, the mass transfer rate between the solvent and the non-solvent was increased by the MXene and larger pore channels were formed in the membranes [31]. The water permeation of the membranes were thus improved owing to these lateral pores.

**Figure 3:**
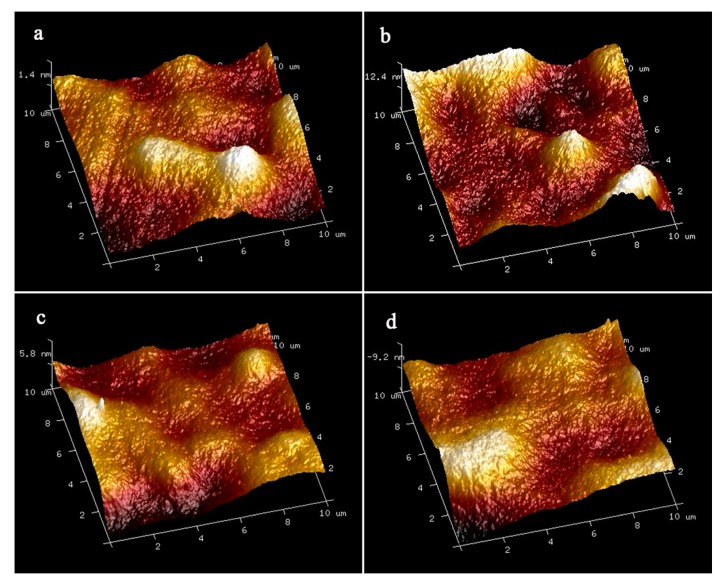
The atomic force microscopy (AFM) images of the prepared membranes: M0 (**a**); M1 (**b**); M2 (**c**); M3 (**d**).

Figure 3 is the atomic force microscopy (AFM) image of the prepared membranes where the brightest area represents the highest point of the membrane surface while the dark area demonstrates the valleys of membrane pores. The surface roughness parameters average roughness (S_a_), root-mean-square of the Z data (S_q_) and height difference between the highest peak and the lowest valley (S_y_) are shown in Table 1. The surface roughness parameters of the composite membranes decreased with the addition of the MXene compared to the neat membrane. One possible reason is that the nanofillers were regularly collocated in membrane. Therefore, many small peaks appeared on the membrane surface instead of large peaks, resulting in a smooth surface of the membrane [32,33]. Reducing the surface roughness was an effective approach to restrict the penetration of foulants into the membrane and adhesion between them [34,35]. All of these contributed to the better antifouling properties of the nanocomposite membranes. 

**Figure 4:**
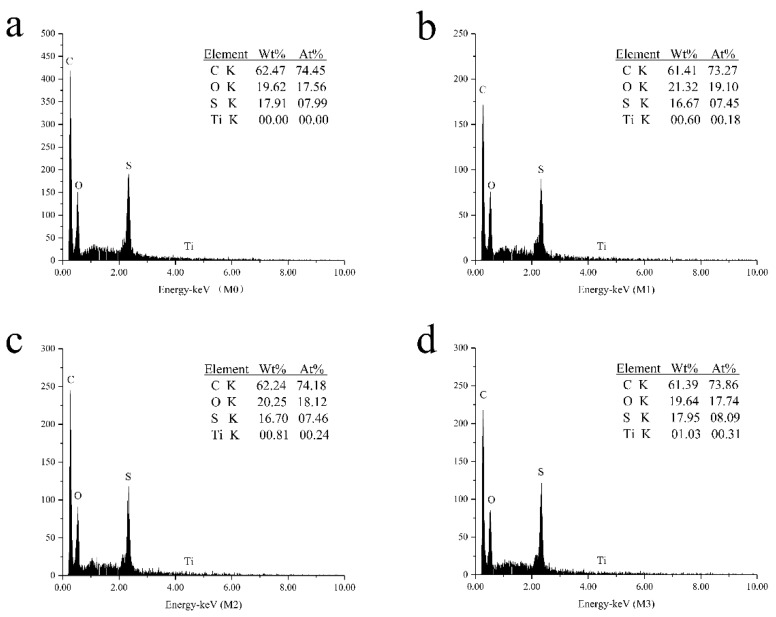
The energy dispersive spectroscopy (EDS) images of the neat and MXene-incorporated membranes ((**a**): M0; (**b**): M1; (**c**): M2; (**d**): M3.).

**Figure 5:**
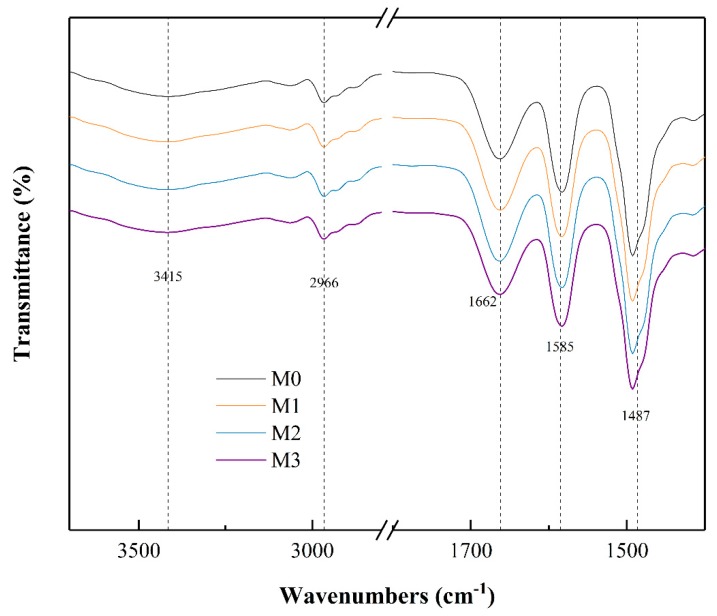
Fourier transform infrared spectroscopy (FTIR) spectra of the prepared membranes.

To assess the existence and quality of the MXene in the membrane, EDS analysis was applied. The spectrums and elements contents are shown in Figure 4, where C, O were mainly sourced from polysulfone and MXene, S was from polysulfone and Ti was from MXene. The presence of Ti confirmed that MXene was successfully embedded into the membrane. FTIR was utilized to analyze the chemical property of the PSF membrane before and after incorporating MXene nanosheets. As is depicted in Figure 5, the absorption bands at 3415, 2966 and 1662 cm^−1^ are corresponding to –OH, –CH and –C = O groups, respectively. The spectra of M3 demonstrated that there was no shift in characteristics vibrational bands with addition of MXene nanosheets, which indicated unchanged chemical structure of PSF and its non-covalent interaction with MXene [7].

The overall porosities of the prepared membranes are shown in Table 2. The mean pore radius presented in Table 2 shows an increase with the concentration of the MXene nanosheets up to 100 mg/L in the water bath. As the concentration of MXene continued to increase, the mean pore radius decreased accordingly. The hydrophilicity effect of MXene increased the solvent and non-solvent exchange during the phase-inversion process which was similar to the graphene oxide [31]. The increased exchange rate led to a higher porosity of the membrane surface. However, when the MXene content increased, the porosity and the mean pore radius of the membrane reduced which is a result of the increasing viscosity of the polymer [7,32].

### 3.2. Thermal Stability and Hydrophilicity

**Figure 6:**
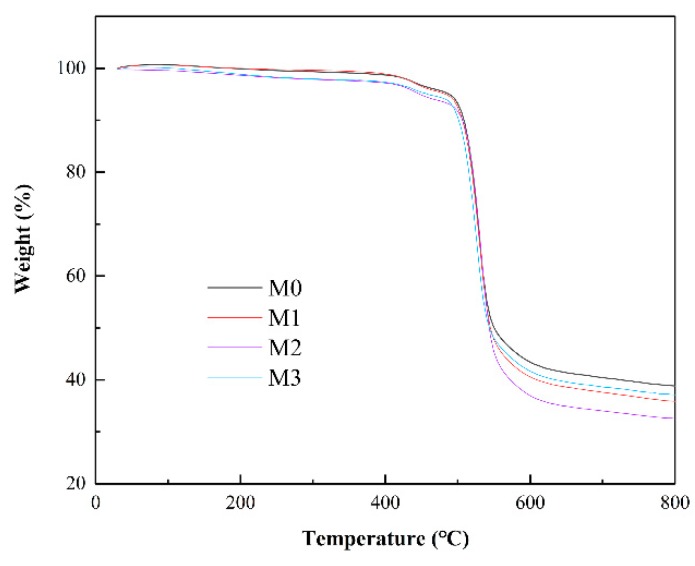
Thermogravimetric analysis (TGA) of the neat and MXene-incorporated membranes.

TGA measurements were conducted to evaluate the thermal stability of the membranes and the results are shown in Figure 6. For all the membranes, there was only one major weight loss between 500 °C and 600 °C which was ascribed to the decomposition of the PSF backbone. Therefore, the nanocomposite membranes possessed good thermal stability as the neat PSF membrane.

Hydrophilicity, one of the most significant parameters for the membrane used for filtration, was closely related to the antifouling properties of the membrane. The water contact angle was considered to be able to reflect the hydrophilicity of a membrane. In general, the lower the water contact angle was, the greater the hydrophilicity of the membrane surface was. Table 3 illustrates that the pristine PSF membrane had the highest water contact angle of 86.9° among all the membranes. With the content of MXene increasing, the water contact angle decreased consistently to 78.4°, indicating the improvement of the composite membranes in hydrophilicity. Higher concentration of MXene nanosheets in the coagulation bath added its content onto the surface of the membrane during the phase inversion step—the hydrophilicity thus improved due to its available hydroxyl groups [7].

Membrane surface charge was another significant parameter of the ultrafiltration membrane [36]. The high surface negative charge could have produced electrostatic repulsion between foulants and membrane surface which is beneficial to antifouling properties [7]. The negative surface charge of the membranes measured at various pH is presented in Table 3. As can be seen from the table, there was an increase in negative zeta potential which was related to the MXene content. The negative surface charge of the membranes was greater than the pristine PSF membrane at all pH values. These were mainly attributed to the hydrophilic groups on membrane surface and led to a better antifouling performance.

### 3.3. Water Permeate and Separation Performance

**Figure 7:**
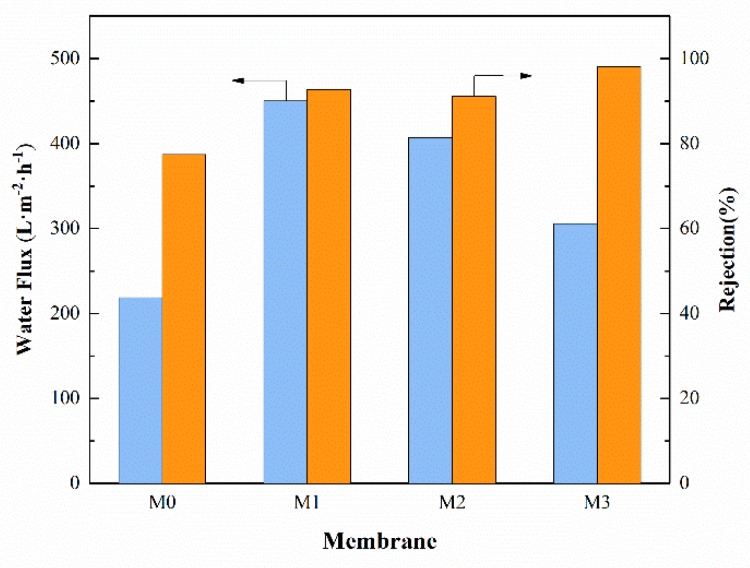
Pure water flux and bovine serum albumin (BSA) rejection of the MXene/polysulfone (PSF) membranes.

To study the effect of the MXene nanosheets on water transport of various membranes, pure water permeability was measured. Figure 7 shows a sharp increase in pure water flux of M1 compared to the pristine PSF membrane M0 under a constant pressure. The water flux of membrane M0 was 218 L·m^−2^·h^−1^ while the flux of nanocomposite membrane M1 reached a peak value of 450 L·m^−2^·h^−1^. Although the water flux of composite membranes decreased when the content of MXene continued to increase, the pure water flux of composite membranes was still higher than that of the pristine membrane. This demonstrates that the increased hydrophilicity of membrane was important in terms of water permeation improvement. Besides, the enhanced porosity and mean pore size also improved the water permeation of the nanocomposite membrane. The ultrafiltration membrane protein rejection was tested under an operating pressure of 0.1 MPa. As is shown in Figure 7, the rejection generally improved as the MXene content increased, and all the composite membranes exhibited high rejection over 90%. The MXene nanosheets surface were composed of F, C, Ti, O, H as well as –OH and –C = O group, and these oxygen-containing groups enabled the MXene composite membranes to be hydrophilic which contributed to the improvement of the water flux of the composite membranes [15]. The enhanced BSA rejection was attributed to enhanced hydrophilicity, mean pore size and the surface charge of nanocomposite membrane resulted from the addition of MXene nanosheets. The increased negative surface charge of the nanocomposite membrane caused by the addition of MXene also led to the repulsive interaction between membrane surface and negatively charged BSA protein and finally provided better protein rejection for the nanocomposite membrane. Therefore, membrane M3 had low water flux but the highest BSA rejection compared to the membrane M1.

### 3.4. Antifouling Performance

Separation performance and usage life which is greatly affected by the membrane fouling are critical to the practical application of membranes. The blockage or plugging of the pores, concentration polarization and cake layer formation are common types of the membrane fouling which have bad influences on the membrane performance [37]. Therefore, a membrane with good quality ought to have the characteristics of high flux, high rejection rate and low fouling tendency. The causes of membrane fouling are relatively complex but the hydrophobicity of the membrane surface does account for the poor antifouling performance of the PSF membranes. To address this problem, hydrophilic material MXene was incorporated into the membrane which was an effective and convenient method to enhance antifouling properties [38,39].

**Figure 8:**
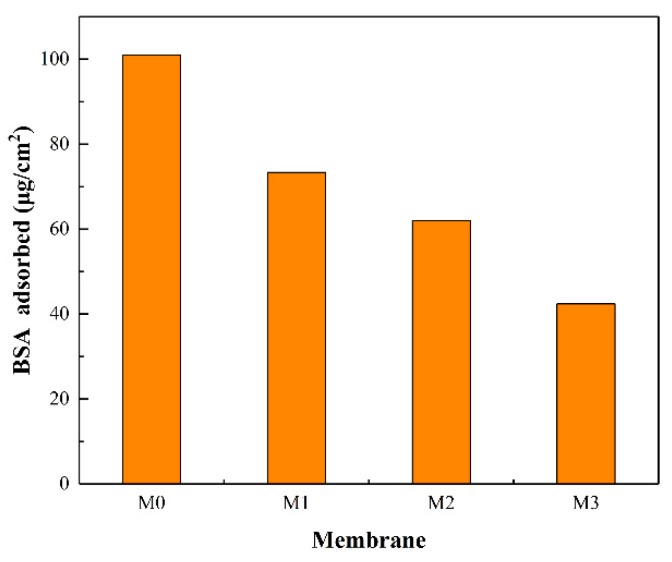
The amount of BSA adsorbed by the prepared membranes.

Figure 8 demonstrates the amount of protein adsorbed from BSA solution to the membrane surface. With the content of the MXene nanosheets increased, the quantity of BSA adsorbed on the composite membrane surface declined, reaching maximum (101.03 μg·cm^−1^) for M0 and minimum (42.39μg·cm^−1^) for M3. The enhanced hydrophilicity reduced the affinity and weakened the interaction between membrane surface and protein, thereby reducing the amount of protein adsorbed [40,41,42].

**Figure 9:**
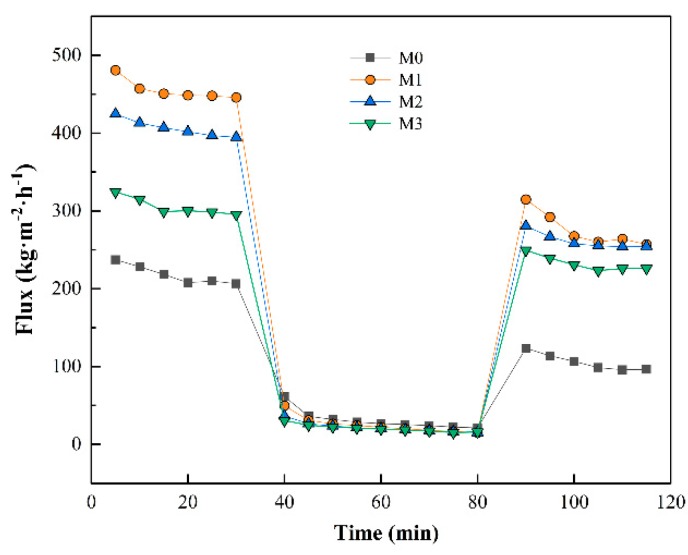
Time-dependent fluctuation of pure water flux and BSA solution flux.

The time dependent flux of the membranes was carried out to evaluate the effects of MXene nanosheets on membrane antifouling properties and reusability. Figure 9 shows the pure water flux before and after protein filtration. A slight decrease in pure water flux was observed for all the membranes during the initial filtration step which resulted from precompaction of the membranes. Then the water flux declined sharply when BSA solution was used as feed. Part of the BSA protein then deposited on the membrane surface, blocking the pores and reducing water permeation of the membrane. All the membranes were then washed with deionized water. The water flux was measured again after physical cleaning so that the restoration ability and contribution of reversible and irreversible fouling in total fouling of every membrane could be measured.

**Figure 10:**
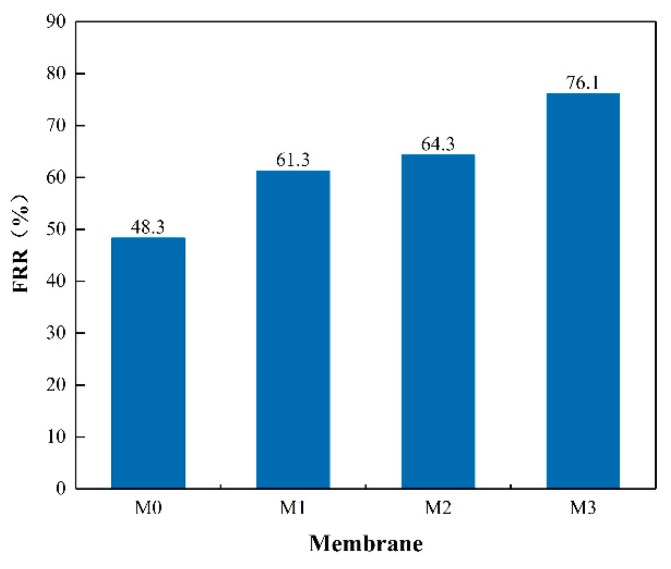
Water flux recovery ratio of the MXene/PSF membranes.

**Figure 11:**
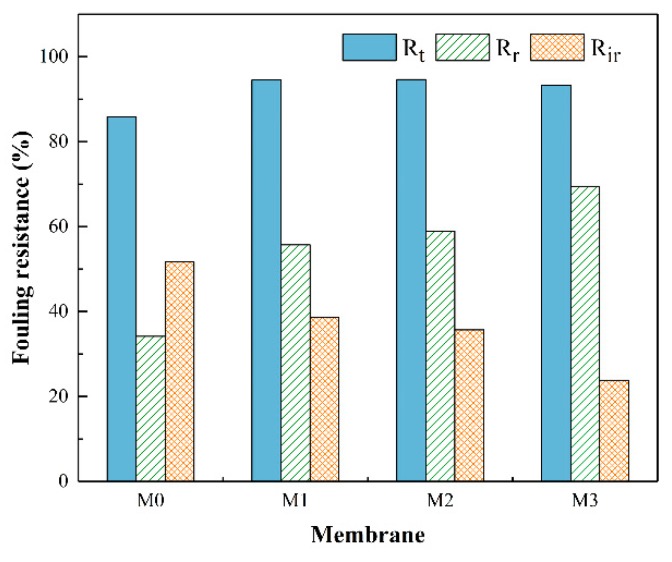
Fouling resistance ratios of the MXene/PSF membranes.

To further evaluate the antifouling properties of the membranes, the flux recovery ratio (FRR), total fouling ratio (R_t_), reversible fouling ratio (R_r_) and irreversible fouling ratio (R_ir_) of each membrane were calculated. The FRR of each membrane is shown in Figure 10 and a higher FRR value usually represents better fouling resistance of the membrane. For the pristine PSF membrane, the FRR was 48.3 percent, which is lower than all the nanocomposite membranes. The membrane M3 possessed the highest flux recovery ratio of 76.1% which is mainly due to its enhanced hydrophilicity. These data indicate that the antifouling properties of the modified membranes were better than the pristine membranes. Fouling can be classified as reversible or irreversible depending on the interaction between the foulants and the membrane surface. Reversible fouling is a result of weak interaction between the fouling particles and the membrane surface which could be easily removed by hydraulically cleaning. Irreversible fouling results from strong interaction of foulants with a membrane surface that cannot be restored by hydraulically cleaning and is harmful to the service life of the membrane [43]. Figure 11 shows the total fouling ratio (R_t_), reversible fouling ratio (R_r_) and irreversible fouling ratio (R_ir_) for all the prepared membranes. As is depicted in Figure 11, the total fouling resistance of the membrane with embedded MXene nanosheets was little higher than the pristine PSF membrane but the irreversible resistance was significantly decreased when MXene content was reduced. The R_ir_ for the membrane M0 was 51.68% and it decreased to 23.87% for the membrane M3. Meanwhile, the R_r_ value tended to be positively related to the MXene content. These results suggested that the foulants were easy to remove because of the enhanced hydrophilicity and low surface roughness of the nanocomposite membrane. Therefore, the nanocomposite membrane had greater antifouling properties than the pristine membrane.

The comparison of different antifouling polysulfone based ultrafiltration membranes is listed in Table 4. The MXene incorporated membrane showed great performance both in water permeation and protein rejection which corresponds to the enhanced hydrophilicity caused by the MXene.

## 4. Conclusions

In this work, MXene incorporated polysulfone membranes were prepared. By dispersing MXene nanosheets into the coagulation bath, the MXene were embedded in the membrane during the phase inversion process. The influences of the embedment of MXene nanosheets on the morphology and performance of the composite membranes were measured by SEM, AFM, EDS, TGA, FTIR, water flux and BSA rejection tests as well as antifouling tests. With the increase of MXene content, the contact angle of the membranes declined from 86.9° to 78.4° and the zeta potential became more negative, indicating an improved hydrophilicity of the composite membranes. All the composite membranes demonstrated a great improvement in water flux and BSA rejection compared to the pristine PSF membrane. The membrane M3 showed a great water flux of 306 L·m^−2^·h^−1^ and the highest BSA rejection of 98%. Besides, the antifouling property of the composite membranes also correspondingly improved, and membrane M3 exhibited the highest flux recovery ratio of 76.1% after physical cleaning. The enhanced antifouling property can be attributed to improved surface smoothness, enhanced hydrophilicity and the more negative zeta potential caused by the MXene nanosheets. Owing to these great improvements, MXene can be considered as a promising material to fabricate antifouling ultrafiltration membranes.

## Figures and Tables

**Table 1:** Surface roughness parameters of the prepared membranes.

Membrane	Roughness Parameters (nm)		
	S_q_	S_a_	S_z_
M0	43.7 ± 5.2	33.8 ± 1.9	261 ± 20.1
M1	33.0 ± 3.7	24.9 ± 3.1	192 ± 19.1
M2	20.8 ± 1.2	16.0 ± 1.8	123 ± 15.4
M3	20.5 ± 1.3	15.5 ± 1.7	138 ± 16.7

**Table 2:** Porosities and mean pore sizes of the prepared membranes.

Membrane	Porosity (%)	Mean Pore Size (nm)
M0	78.5	29
M1	79.4	41
M2	78.8	39
M3	74.4	36

**Table 3:** Water contact angle and zeta potential of all the prepared membranes.

Membrane	Water Contact Angle (°)	Zeta Potential (mV)		
		pH = 4	pH = 5	pH = 7
M0	86.9	−2.34	−6.25	−14.17
M1	82.6	−2.84	−7.15	−15.38
M2	79.3	−2.96	−8.97	−18.23
M3	78.4	−3.16	−10.14	−21.01

**Table 4:** Comparison of different antifouling polysulfone based membranes.

Membrane	Additive	Water Flux (L·m^−2^·h^−1^)	Rejection (%)	Reference
PSF/MWNTs	Oxidized MWNTs	70.7	61.9	[44]
PSF/iGO	Isocyanate GO	130	95	[33]
PSF/SiO_2_-GO	SiO_2_-GO	376	98	[45]
PSF/ONC	Oxidized nanocarbon	307	97.6	[7]
PSF/MXene	MXene nanosheets	306	98	This work
